# 伴顽固性胸水并发乳糜胸的华氏巨球蛋白血症1例

**DOI:** 10.3760/cma.j.cn121090-20241229-00602

**Published:** 2025-08

**Authors:** 雨涵 郑, 迪 杨, 笑 严

**Affiliations:** 1 宁波大学附属第一医院血液科，宁波 315010 Department of Hematology, The First Affiliated Hospital of Ningbo University, Ningbo 315010, China; 2 华中科技大学附属协和医院血液科，武汉 430022 Department of Hematology, Union Hospital, Tongji Medical College, Huazhong University of Science and Technology, Wuhan 430022, China

患者，女，45岁，因干咳、气促伴间断低热2周（体温最高37.4 °C）于2023年1月就诊于襄阳市中心医院，查胸部超声示左侧大量胸腔积液，未见明显占位。予胸腔穿刺置管，引流血性液600 ml，此后引流液转为黄色清亮，400～500 ml/d。患者完善PET-CT检查提示多部位代谢偏高，纵隔及双侧肺门区多发小淋巴结；右侧心膈角区条片状软组织密度影，糖代谢轻度增高，考虑多为增厚胸膜或炎性病变。行右颈部淋巴结活检，病理考虑淋巴浆细胞淋巴瘤。

2023年2月患者至华中科技大学附属协和医院住院治疗。入院时感胸闷、气促，浅表淋巴结未及肿大，左肺呼吸音低，右肺呼吸音粗，未闻及明显啰音。入院后完善相关检查，B型超声波检查示左侧大量胸腔积液；IgM 34.6 g/L；M蛋白：IgM-κ型M蛋白血症。淋巴结病理结果示：（右颈部淋巴结）淋巴浆细胞淋巴瘤。免疫组化染色示肿瘤细胞：CD19（+），CD20（+），CD79a（+），CD3（−），LEF1（−），CD23（−），CD35（−），CD43（−），CD5（−），CD10（−），IUI1（浆样细胞+），BCL2（+），CD30（−），Ki-67（8％+），CD138（浆样细胞+），TDT（−），κ（浆样细胞+），λ（浆样细胞−）；原位杂交检测EB病毒示EBER（−）。基因检测结果示：MYD88基因突变阳性，Exon5发生突变，突变类型为：c.794T>C(P.L265P)。骨髓细胞学可见形态异常的淋巴细胞。骨髓流式细胞术：有核细胞中，1.79％为表型异常的单克隆成熟小B细胞，表达CD19、CD20、κ、CD79b、BCL-2，部分表达CD180、CD38，不表达λ、CD5、CD10、CD23、CD34、FMC7、Ki-67、CD103、CD138，前向散射光和侧向散射光较小。骨髓活检：肿瘤细胞约占有核细胞50％；免疫组化结果与上述淋巴结活检结果一致。胸腔积液流式细胞术：有核细胞中，5.53％细胞表达CD19、CD20、CD79b、BCL-2，κ/λ比值增高，为4.2；不表达CD5、CD10、CD200、FMC7、CD34、CD38、Ki-67、CD103、CD138，可疑为表型异常的单克隆成熟小B细胞。综合以上检查，患者确诊为淋巴浆细胞淋巴瘤/华氏巨球蛋白血症（WM），WM预后评分2分，中危组。患者拒绝行胸腔积液基因检测。

入院后患者胸腔积液引流不畅，更换胸腔引流管后持续引流液体500 ml/d，最初为血性，后转为黄色清亮。考虑到患者为WM合并顽固性恶性胸腔积液，有髓外病变。予BR方案（苯达莫司汀90 mg/m^2^，第1、2天；利妥昔单抗375 mg/m^2^，第1天）化疗1个疗程，患者胸腔积液明显减少，为100～200 ml/d。

患者出院后每天进食约1 000 ml牛奶和丰富的肉类食品。化疗后第4天患者进食大量鲜牛奶及鸽子汤后引流出乳白色胸腔积液400 ml，伴皮疹、高热，急诊入我院。予抗过敏、抗感染治疗后，患者皮疹明显消退，体温恢复正常。C反应蛋白轻度升高，胸腔积液多次培养和病原体二代测序均为阴性，乳糜试验阳性，考虑乳糜胸。纯素食3 d后，患者胸腔积液转为黄色清亮，自行出院。

出院1周后患者再次感到心悸、胸闷，超声波查示左肺大量胸腔积液，持续胸腔引流血性液体800 ml/d。再次予BR方案化疗，利妥昔单抗治疗过程中患者出现畏寒、发热，自诉无法耐受，要求停用利妥昔单抗，仅使用苯达莫司汀。化疗后患者胸腔积液一度较前增加，且因进食油腻食物再次出现血性乳糜胸。低脂饮食后乳糜胸好转，胸腔积液引流量150 ml/d。考虑患者第2个疗程单药治疗，疗效不佳，2023年4月改用泽布替尼160 mg每日2次口服。半个月后IgM降至5.5 g/L；骨髓中肿瘤细胞占比下降至20％，骨髓流式细胞术显示肿瘤细胞下降至0.27％。考虑泽布替尼疗效较好，口服至今。2024年5月随访，患者无自觉症状，一般情况良好，无明显胸腔积液，血常规示轻度白细胞减少。

